# Effect of the QM Size, Basis Set, and Polarization on QM/MM Interaction Energy Decomposition Analysis

**DOI:** 10.1021/acs.jcim.2c01184

**Published:** 2023-01-20

**Authors:** Álvaro Pérez-Barcia, Gustavo Cárdenas, Juan J. Nogueira, Marcos Mandado

**Affiliations:** † Department of Physical Chemistry, University of Vigo, Lagoas-Marcosende s\n, ES-36310-Vigo, Galicia, Spain; ‡ Department of Chemistry, Universidad Autónoma de Madrid, 28049, Madrid, Spain; ¶ Institute for Advanced Research in Chemistry (IAdChem), Universidad Autónoma de Madrid, 28049Madrid, Spain

## Abstract

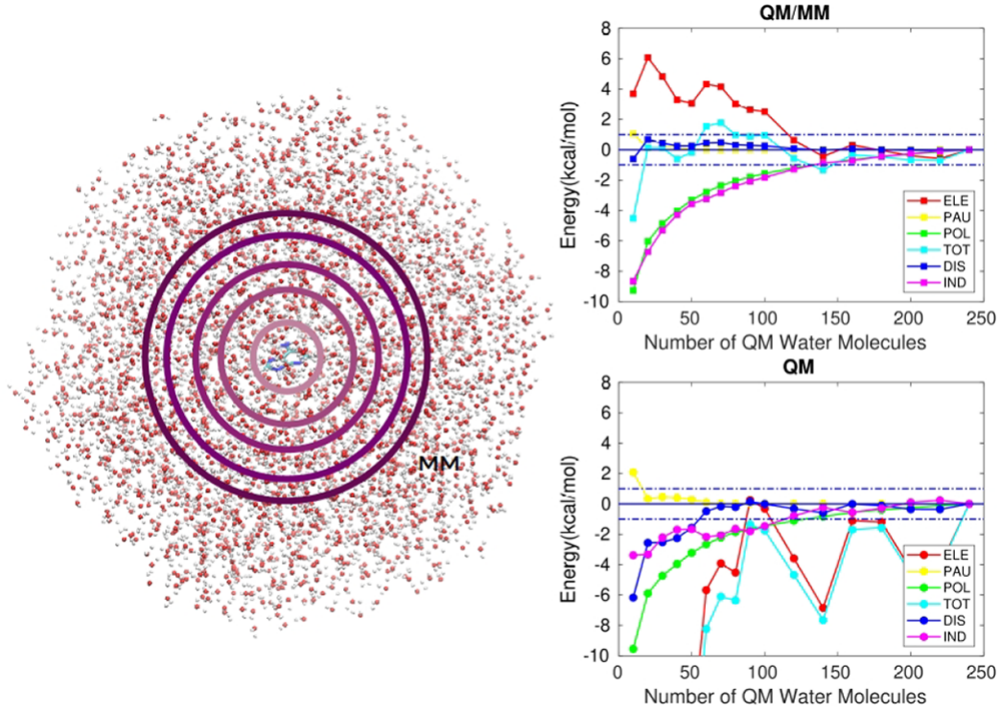

Herein, an Energy Decomposition Analysis (EDA) scheme extended to the framework of QM/MM calculations in the context of electrostatic embeddings (QM/MM-EDA) including atomic charges and dipoles is applied to assess the effect of the QM region size on the convergence of the different interaction energy components, namely, electrostatic, Pauli, and polarization, for cationic, anionic, and neutral systems interacting with a strong polar environment (water). Significant improvements are found when the bulk solvent environment is described by a MM potential in the EDA scheme as compared to pure QM calculations that neglect bulk solvation. The predominant electrostatic interaction requires sizable QM regions. The results reported here show that it is necessary to include a surprisingly large number of water molecules in the QM region to obtain converged values for this energy term, contrary to most cluster models often employed in the literature. Both the improvement of the QM wave function by means of a larger basis set and the introduction of polarization into the MM region through a polarizable force field do not translate to a faster convergence with the QM region size, but they lead to better results for the different interaction energy components. The results obtained in this work provide insight into the effect of each energy component on the convergence of the solute–solvent interaction energy with the QM region size. This information can be used to improve the MM FFs and embedding schemes employed in QM/MM calculations of solvated systems.

## Introduction

Simulating polar environments accurately is challenging at the quantum level but crucial in order to reproduce experimental data. For instance, many biochemical processes take place under strong polar conditions, where not only the closest surrounding solvent molecules take part actively but more distant molecules also influence the process by creating a non-negligible electrostatic potential that changes the intra- and intermolecular interactions established.^1−5^ The long-range character of these electrostatic interactions requires the consideration of a large number of atoms in the simulations, including a significant part of the environment. Thus, application of pure quantum mechanical (QM) methods is not feasible and these must be replaced by hybrid quantum mechanics/molecular mechanics (QM/MM) methods.^6−8^ Pure molecular mechanics (MM) methods can also be employed to deal with systems including thousands of atoms, but the price to pay in terms of accuracy is sometimes too high, especially when dealing with processes where electrons have to be explicitly described. Particularly delicate are the studies of chemical transformations that involve, for instance, bond breaking or formation, photochemical activation, or strongly correlated systems. In these situations, a quantum mechanical treatment is strongly recommended or even mandatory. More accurate approaches employed to take into account environmental effects are hybrid QM/QM techniques, which combine two levels of QM theory, such as the polarizable density embedding^9^ and frozen density embedding.^10^ However, they are not extensively used in the simulation of large systems because of their computational cost. When the environment to be modeled is isotropic, e.g., solvents, continuum approaches are a very good alternative because of their low computational cost and ability to describe polarizing effects.^11^ However, in many cases, explicit interactions, such as hydrogen bonding, are not properly described.

The fundamental idea behind QM/MM methods is the partition of the full system into two regions: a small one, the target, and a larger one, the surroundings, which are described at QM and MM levels, respectively. Obviously, the QM region includes the most important part of the system, where the process takes place, whereas the MM region contains the remaining atoms. Therefore, the full system Hamiltonian gets separated into three parts: QM, MM and QM-MM ones, the latter accounting for the interactions established at the interface between both regions. It is for this last term in particular, where the different schemes developed for the implementation of QM/MM methods differ. The QM/MM approaches^12^ may be grouped within either the additive scheme,^13,14^ where a term describing the interaction between the QM and MM regions is explicitly constructed, or the subtractive scheme,^15,16^ where the explicit construction of a QM-MM interacting term is avoided. The most popular subtractive schemes are the integrated molecular orbital molecular mechanics (IMOMM)^15^ method and its subsequent improvements, all of them belonging to the family of “our own *n*-layered integrated molecular orbital and molecular mechanics” (ONIOM) methods^17^ by Morokuma et al.^18^ The original formulation of the subtractive scheme computed the interaction between the different layers only at classical level by using a classical force field (FF). However, later formulations of the subtractive scheme allow a more accurate description, as will be explained below.

Even though the subtractive scheme is computationally simpler, the additive scheme is often preferred due to its higher flexibility. In the additive schemes, the interaction between the QM and the MM regions may be treated at four different levels: using mechanical,^15,16^ electrostatic,^13,19−21^ polarizable,^22−24^ or flexible boundary^25,26^ embedding schemes. The mechanical embedding treats the interface interactions at the MM level, which does not allow including the polarization of the wave function of the QM region by the MM charges, a factor extremely important for systems subjected to strong polar environments. The electrostatic embedding is by far the most employed one due to its good compromise between accuracy and computational simplicity. In this approach, the polarization of the QM region by the electrostatic potential created by the MM atomic charges is accounted for. In the polarizable embedding, mutual polarization between the QM and MM regions is taken into account, self-consistently or not. In addition to the electrostatic interaction with the MM atomic charges, the interaction between the induced dipole moments of the MM region and the electric field created by the QM region is introduced in the Hamiltonian. For this reason, a polarizable FF must be employed to describe the classical region. It must be recalled that the MM atomic charges in electrostatic FFs are fixed and different than those of polarizable FFs since they have been parametrized without atomic dipoles. For obvious reasons, polarizable embedding schemes are more computationally demanding than the electrostatic ones.

Finally, flexible boundary embedding schemes are even more sophisticated approaches based on the principle of electronic chemical potential equalization, allowing both partial charge transfer and self-consistent polarization between the QM and MM regions.^25,26^ Although these schemes improve the accuracy, they also increase the computational cost significantly. The higher computational cost of polarized embedding schemes, combined or not with the flexible boundary approach, is the main reason the electrostatic schemes are still preferred for most dynamic and static simulations.

As mentioned above, original subtractive ONIOM methods are based on the mechanical embedding scheme (ONIOM-ME), and thus, its main drawback in QM/MM calculations is the lack of polarization effects of the QM wave function by the MM region. This can be circumvented by adding the MM electrostatic potential in the QM calculation, which, in the context of ONIOM methods, is known as electronic embedding (ONIOM-EE). To avoid the computation of the electrostatic interactions between layers twice, this term is also computed classically and subtracted from the total energy. The accuracy improvement introduced by the ONIOM-EE method obviously entails a rise in computational cost with respect to ONIOM-ME.

Either for additive or subtractive schemes a crucial step in the setup of QM/MM calculations involving strong polar environments is the selection of the QM region size. If the target system involves small or medium size solutes (formed by a few tens of atoms) surrounded by solvent molecules, the QM region can be enlarged by just transferring more solvent molecules from the MM region. This way, nonbonded interactions are included in the QM-MM interface, thus avoiding one of the main problems of the QM/MM methods, namely, the truncation of the QM region through covalent bonds and the subsequent, often required, redistribution of charges within the atoms of the interface. Then, the crucial question here is how many solvent molecules must be incorporated into the QM region to accurately describe the intermolecular solute–solvent interactions? This work addresses this question from a rigorous point of view.

Previous works have already dealt with the problem of the QM size for the study of different properties, such as absorption spectra,^27−30^ excitation energies,^31^ charge transfer processes,^32^ NMR shieldings,^33^ proton transfer,^34,35^ enzyme catalysis,^36−39^ and interaction energies.^40^ In the particular case of activation energies and/or reaction energies, a strong dependence of the optimum QM region size with the QM level and MM embedding scheme employed has been found. Thus, a relatively small number of atoms (lees than 100) is required when semiempirical methods are employed,^35,36^ whereas a much larger number of atoms in the QM region is required when DFT methods are applied,^37−39^ even more than 1000.^34^ This slow convergence of reaction energies with the QM region size in enzymatic reactions has been related to the charge transfer between the active site and the surrounding protein, which cannot be addressed by improving the MM force fields. A faster convergence is obtained when going from mechanical to electrostatic embedding^31^ and with the use of flexible boundary embedding schemes.^25,26^

However, the problem of the QM region size convergence was never approached for the study of long and short-range solute–solvent interaction energies, at least using a rigorous QM energy decomposition analysis (EDA) reformulated under the action of a MM solvent environment. This will be referred to as QM/MM-EDA. The EDA scheme employed here was developed and implemented some years ago at the QM level.^41,42^ Its extension to hybrid QM/MM methods was recently presented and applied to the study of lipid membrane permeability.^43^ Herein, this QM/MM-EDA scheme is employed to investigate the QM size required to accurately describe long- and short-range interactions between an ionic solute and a highly polar solvent. As paradigm of strong polar solvents, we have chosen water, whereas cationic, anionic, and zwitterionic solutes have been chosen in order to account for the three different types of ionic molecules. A previous study of the effect of the QM region size on intermolecular interaction energies calculated with QM/MM methods was carried by Fox et al.^40^ Although focused on the same issue, two important differences between the present work and that performed by Fox et al. should be remarked. The first one is that Fox et al. compared, for different sizes of the QM region, the interaction energy between fragments defined within the QM region but polarized by an MM electrostatic embedding, whereas in our work one fragment (the solute) is defined within the QM region but the other fragment (the solvent) contains all the solvent molecules included in the QM and MM regions. Thus, the interactions of the solute with the solvent molecules included in the MM regions are also included in the interaction energy. The second important difference is that Fox et al. only focused on the total interaction energy, whereas the different interaction energy terms (electrostatic, Pauli repulsion, induction and dispersion) are also analyzed in our study through the QM/MM-EDA scheme mentioned above. Additionally, there have been studies where an EDA scheme has been applied to the study of interactions between QM and MM regions and where the influence of the QM or MM region size on these interactions has been assessed.^44−46^ The important difference of these works with respect to our work is that the fragments considered always belonged to one of the regions (QM or MM), whereas we consider QM/MM hybrid fragments in our QM/MM-EDA scheme.

It must be remarked that understanding the role of each energy term on the convergence of the interaction energy with the QM region size in QM/MM calculations will help to correct deficiencies in the MM FFs and embedding schemes. It has been found very recently that, in the permeation of chlorinated dioxins through lipid membranes, the dispersion energy is the term with the slowest convergence and not the electrostatic energy as could be expected in general.^47^

## Theoretical Background: QM/MM-EDA

In this section, the EDA employed in this work is first introduced at QM level, and then the particular case of a hybrid QM/MM level is addressed. The equations at QM level were previously introduced elsewhere.^41,42^ The readers are referred to these works to extend their knowledge on this perturbational EDA scheme based on electron deformation densities.

For an interacting quantum system formed by two closed-shell fragments A and B, the interaction energy is defined as the difference between the AB complex energy and the energies of the isolated fragments at the complex geometry, as indicated in eq 1 by the superscript AB.
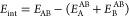
1Due to the use of finite basis sets, the fragment energies in eq 1 are also calculated with the basis set of the complex in order to correct the basis set superposition error following the counterpoise method.^48^ The AB complex energy may be written in terms of the one-electron density, the one-electron density matrix and the exchange-correlation density,
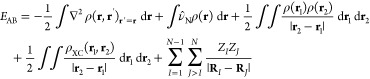
2In eq 2, ρ(**r**) and ρ(**r**, **r**′)_**r**′=**r**_ represent the one-electron density and density matrix, respectively, ρ_XC_(**r**_1_, **r**_2_) represents the exchange-correlation density, ν̂_N_ represents the nuclei potential operator, and **r** and **R** represent the electron and nuclei positions, respectively. Operators, densities and density matrices may be decomposed into terms from a hypothetical noninteracting system (unperturbed fragments) and the interacting terms (eqs 3–6). Thus, the nucleus–nucleus repulsion energy term and the nuclei potential operator are given by,
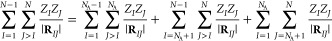
3
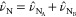
4where *N*_*A*_ and *N*_*B*_ represent the number of nuclei of fragments A and B, respectively, and **R**_*IJ*_ = **R**_*I*_ – **R**_*J*_. The one-electron and exchange-correlation densities are given by,
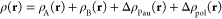
5
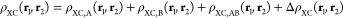
6where the contributions from the Pauli repulsion and the electron polarization, *Δρ*_Pau_(**r**) and *Δρ*_pol_(**r**), have been included separately in eq 5 as well as the interfragment exchange and polarization, ρ_X,AB_(**r**_1_, **r**_2_) and *Δρ*_XC_(**r**_1_, **r**_2_), in eq 6. The first two terms in eqs 5 and 6 correspond, respectively, to the one-electron and exchange-correlation densities of the unperturbed fragments. An equivalent partition to that of eq 5 applies to the one-electron density matrix.

Unperturbed fragment potentials, *v̂*_*A*_ and *v̂*_*B*_, can be also defined by merging the nuclei potential and the electron potential operators,
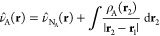
7
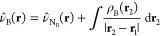
8Thus, introducing eqs 3–8 into eq 2, removing the energies corresponding to the unperturbed fragment energies, and grouping those with the same physical origin, the interaction energy gets decomposed into electrostatic (elec), exchange (exch), repulsion (rep), and polarization (pol) energies,
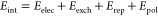
9The explicit expressions for these energy contributions are,
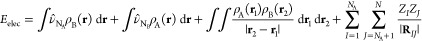
10
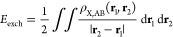
11
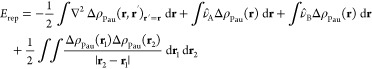
12
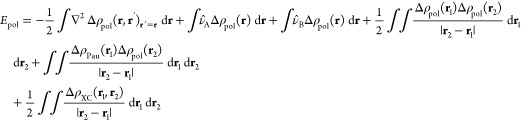
13Even though repulsion is a functional of *Δρ*_*Pau*_ and exchange is a functional of ρ_X,AB_, both arise from the same fundamental quantum mechanical principle, the Pauli exclusion principle. Thus, these energy terms arise from the Fermi correlation between same-spin electrons of different unperturbed fragments, so that they are generally merged into one term called Pauli energy, *E*_Pau_. On the contrary, polarization includes contributions from induction and dispersion forces, and can be separated exactly into induction and dispersion energies at second-order perturbation theory (PT) level.^49^ Therefore, combining our EDA scheme with second-order PT, the induction energy can be extracted from the rest of polarization.^42^ In the Rayleigh–Schrödinger PT (RSPT), the induction energy^49^ and the first-order correction to the electron density,^42^ the one required for the calculation of the second-order energy, are given by eqs 14 and 15,
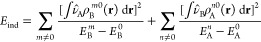
14
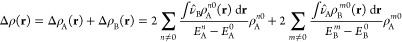
15where the notation ρ_A_^*n*0^ and ρ_B_^*m*0^ has been introduced to denote induced transition densities within fragments A and B from ground state configuration 0 to excited state configurations *m* or *n*. In the equations above, denominators include the energies corresponding to these transitions.

As explicitly shown in eq 15, the first-order correction to the electron density is split into contributions from fragments A and B. This electron density correction represents the polarization density at second-order, so that substituting eq 15 in eq 13 the induction energy may be extracted. However, it is more straightforward to define the charge-induction energy, *E*_ch-ind_, as the sum of the second and third terms of eq 13 and work with it from now on,
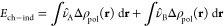
16To extract the induction energy (eq 14) from the charge-induction energy (eq 16), three straightforward steps must followed. First, introduce eq 15 into eq 16,
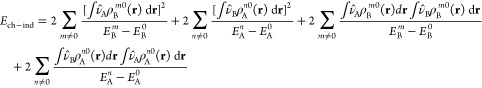
17Second, identify *E*_ind_ (eq 14) as one-half of the first two terms of eq 17 and reorder the terms,
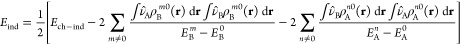
18and third, replace back the definition of first-order density (eq 15) to obtain the final expression for *E*_ind_,
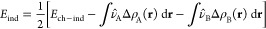
19which corresponds to one-half of the charge-induction energy minus the intrafragment energies. Equation 19 may be rewritten as,
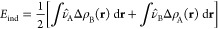
20which reproduces the classical result where half of the induction energy is wasted in the induction process.

Once induction is obtained, the rest of polarization corresponds exclusively to dispersion at second-order PT. However, if the intermolecular interaction is large, then second-order PT is no longer a good approximation, and higher order corrections are required. These higher order corrections introduce more induction and dispersion but also mixed terms, so that the partition of polarization into pure induction and dispersion contributions is not reliable.

In the QM/MM-EDA scheme employed in this work, the potential created by the MM region in the QM region is introduced in the calculation of the total interaction energy and energy components. Notice that the center of the intermolecular interaction must be described at QM level, and so, the fragments must be partially, or even completely, described quantum mechanically. For instance, in the present study the solute molecule is fully described at QM level whereas the solvent is partially described at QM and MM levels. In QM/MM- EDA calculations with an electrostatic embedding,^43^ the MM potential at the QM region, *V̂*_*FF*_(**r**), includes only the potential created by fixed atomic charges. The form of this potential is given by,
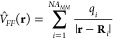
21where *q*_*i*_ and **R**_*i*_ represent the atomic charges and their positions and *NA*_*MM*_ denotes the number of atoms in the MM region. In our QM/MM-EDA scheme, *V̂*_*FF*_(**r**) is merged with the nuclei potential of the corresponding fragment so that it is included in the calculation of the electrostatic, repulsion and polarization energies (eqs 10, 12, and 13). In the present study, where the MM region is fully included within the solvent (fragment B), only the nuclei potential of this fragment is replaced by the QM/MM potential.
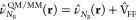
22

It must be noticed that the MM atomic charges also interact electrostatically with the nuclei of the solute molecule. Besides the replacement of ν̂_*N*_B__ by ν̂_*N*_B__^QM/MM^(**r**) in eq 10, the energy of this interaction must be added to obtain the electrostatic energy at QM/MM level.
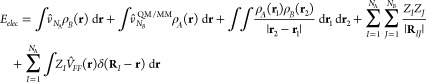
23

In addition to the polarization of the QM region by the MM region, which was accounted for by QM/MM energy calculations performed in previous works, this QM/MM-EDA scheme also introduces explicitly the MM potential in the EDA step, providing a correct interaction energy decomposition, which is not obtained otherwise.

On the other hand, in an electrostatic embedding including induced atomic dipole moments (polarizable FF) *V̂*_*FF*_(**r**) incorporates the potential created by the induced dipoles in the QM region. The form of the potential is now given by,
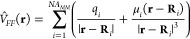
24where μ_*i*_ represents the atomic dipole. Herein, each dipole is replaced by a pair of opposite induced charges, δ_*i*_^+^ and δ_*i*_^–^, located on the atom, separated by a distance **d**_*i*_ and aligned with the dipole vector. eq 24 is then rewritten as,
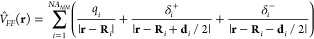
25|**d**_*i*_| must be much shorter than the shortest distance between a QM and a MM atom, so that the values of the MM potential within the QM region do not change when going from eq 24 to eq 25. With this very small value of **d**_*i*_, the induced charges have to be large in order to reproduce the values of the induced dipole moments. These large induced charges yield a huge total electrostatic energy at the MM region due to the intraatomic electrostatic interactions between the positive and the negative charge of each pair. This interaction is introduced by the model but has no physical sense. However, neither the interaction energy between the solute and the solvent nor the later EDA is affected by the model of induced charges as only the MM electrostatic potential at the solute region is taken into account for these calculations.

## Computational Details

The systems here studied consist on three ionic solutes surrounded by a water environment; the ammonium cation, zwitterionic glycine and the formate anion. As we intend to investigate the full dynamic process instead of a static situation, previous to the QM/MM calculations, molecular dynamics (MD) simulations were carried out to generate an ensemble of geometries to perform a subsequent statistical analysis of the different interaction energy terms. For the MD simulations the solvent molecules were placed on a truncated octahedron with a distance of 22 Å separating any molecule from the edge of the simulation box. MD simulations were run with AMBER20,^50^ modeling solutes with the GAFF(GAFF2),^51^ and solvent molecules with the TIP3P^52^ and POL3^53^ water models as implemented in the PMEMD.CUDA^54,55^ and SANDER modules, respectively. Replacing the TIP3P electrostatic FF by the POL3 polarizable FF allowed to evaluate the effect of introducing atomic dipoles in the MM region on the optimal QM region size. A minimization of 2000 steps switching from steepest descent to conjugated gradient algorithms after 10 steps was followed by heating to room temperature during a 0.5 ns thermalization step in the NVT ensemble employing a Langevin thermostat with a collision frequency of 1 ps^–1^. A classical MD simulation lasting 10 ns with a time step of 2 fs in the isothermal–isobaric (NPT) ensemble was then performed to obtain the set of geometry snapshots, each of them saved every 10 ps. The Berendsen barostat^56^ was used at pressure 1 bar and a relaxation time of 2 ps. Periodic boundary conditions were used during the simulation. The charge neutrality required by the Ewald particle summation^57,58^ was achieved by including Na^+^ and Cl^–^ as counterions^59^ for the formate anion and the ammonium cation, respectively. The cutoff for both Lennard-Jones potential and the real-space Ewald particle summation was set to 12 Å. Bond distance involving hydrogen atoms were restrained by the SHAKE algorithm.^60^

In order to characterize the optimal QM region size for QM/MM calculations, a single snapshot was randomly selected from the last 5 ns of the MD simulation. Then, the QM/MM calculations were performed with Gaussian16,^61^ using the M062X^62^ functional and the cc-pVDZ basis set, later increasing the basis set to a cc-pVTZ for the whole system.^63−67^ The QM region was defined with the solute at its center and the number of closest water molecules was increased from 10 to 240, with the remaining solvent molecules included as point charges assigned by the TIP3P model. The MoBioTools toolkit^68^ (https://github.com/mobiochem/MoBioTools) was used for the definition of each region. To perform the EDA, three single point QM/MM calculations were carried out for each system without periodic boundary conditions but including the whole environment in the MM layer, corresponding to the full system (QM region and MM point charges) and the fragments on which to compute the interaction energy. Fragment 1 consisted of the QM solute in the presence of the basis set of the QM water molecules, whereas fragment 2 included all QM water molecules as well as the basis set from the solute surrounded by the MM electrostatic embedding. Basis functions of absent atoms are aimed at accounting for the basis set superposition error (BSSE) by means of the counterpoise correction (CPC).^69,70^ Finally, the EDA-NCI program (https://github.com/marcos-mandado/EDA-NCI) was used to perform the energy decomposition analysis. The full theoretical background of the QM/MM-EDA scheme applied in this work^41,42^ was included in the previous section. To introduce vibrational sampling and compare the energy components obtained with both the TIP3P and POL3 FFs, 100 snapshots were selected from the last 5 ns of the MD simulation each 50 ps so that they were not correlated, and thus, a broad region of the configurational space is covered by the sampling protocol. The induced atomic dipoles of the MM region are directly taken from the MD simulation, as are the fixed atomic charges. It is important to highlight that, within this electrostatic QM/MM embedding scheme including induced atomic dipoles, the mutual polarization between the solvent and the solute is computed at classical level during the MD simulation, but then, the induced dipoles of the solvent are fixed in the subsequent QM/MM computations of the interaction energy.

## Results and Discussion

In this section, the convergence of solute–solvent interaction energies and its different components is evaluated for the three different solutes, namely, the ammonium cation, zwitterionic glycine and the formate anion. Given the long-range nature of electrostatic interactions, the polarity of each studied system is likely to impact the convergence of the interaction energy and its components. In this sense, one can tackle the problem of QM/MM convergence either by improving the description of the QM region or that of the MM embedding. For this reason, the initial QM calculations performed with the M062X functional and the cc-pVDZ basis set are supplemented by the larger cc-pVTZ basis. Regarding the MM region, a polarizable FF (POL3)^53^ is introduced to further improve the description of the MM water molecules provided by the TIP3P electrostatic model.

### Interaction Energy Convergence with QM Region Size

**Figure 1:**
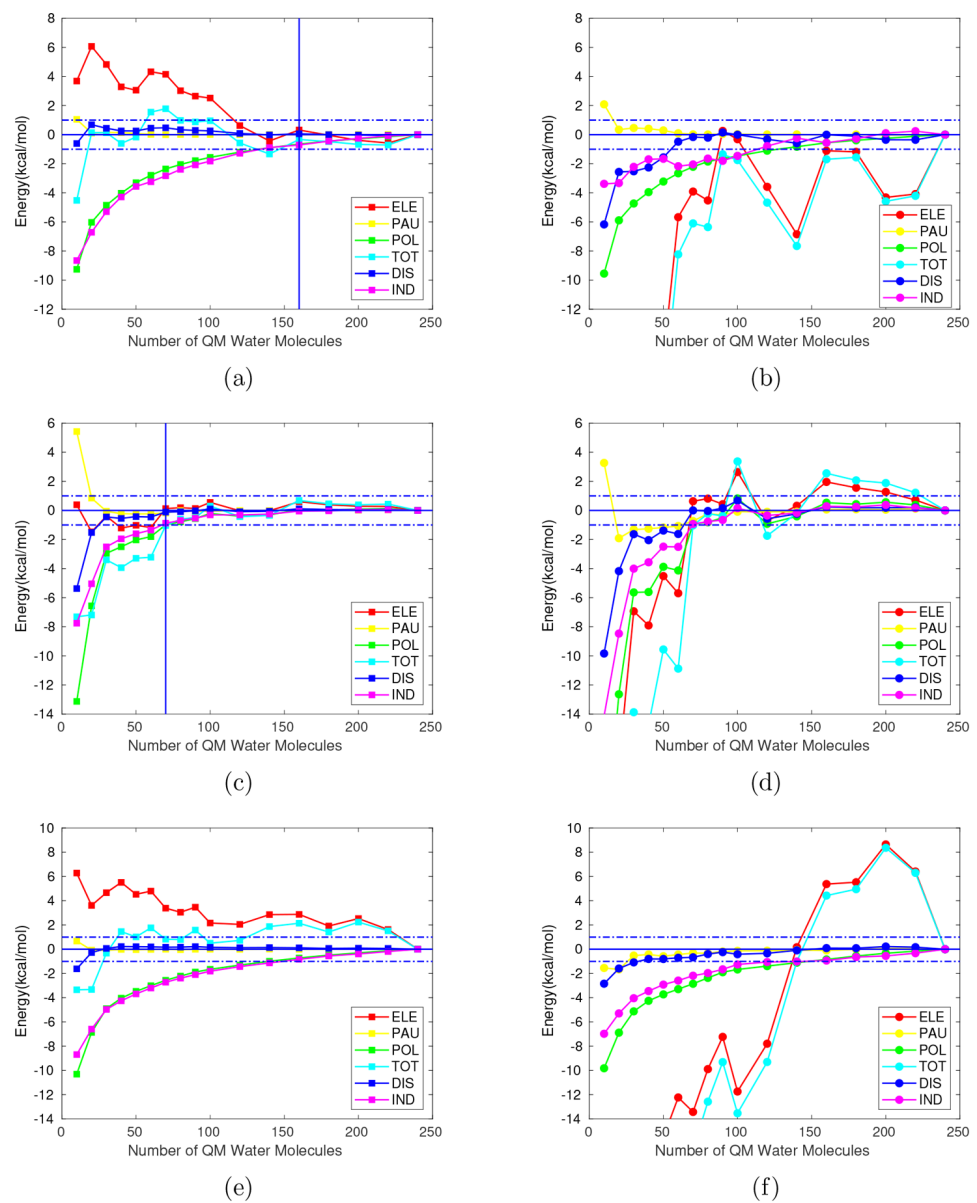
Interaction energy components in kcal/mol (M062X/cc-pVDZ) relative to the limit value for a QM region with 240 water molecules (*E*_*i*,*QM*/*MM*_^240^ and *E*_*i*,*QM*_^240^) for different sizes of the QM region within an MM (TIP3P) electrostatic embedding (left) and without it (right). Ammonium (top), glycine (middle) and formate (bottom). Horizontal dashed lines: deviation of ±1 kcal/mol from *E*_*i*_^240^, where *i* represents each component of the interaction energy. Vertical lines: point of convergence of all the energy components within the ±1 kcal/mol range.

Initially, the behavior of the total interaction energy and its different components will be analyzed as a function of QM region size at the M062X/cc-pVDZ level for a single snapshot selected from the last 5 ns of the MD trajectory. As the surrounding MM potential will impact significantly the electron density distribution, specially for QM regions of small size, the effect of the MM region on the obtained results is also evaluated. For each solute, the deviations of the interaction energy components with respect to the values computed for a QM region containing 240 water molecules (reference values can be seen in Table 1) is calculated for various QM region sizes. An identical procedure is performed in the absence of the MM electrostatic embedding with results presented in Figure 1 (for full scale representations of the total and electrostatic interaction energy components check Figure S1 in the Supporting Information). A QM region containing 240 water molecules corresponds to quasi-spheres of radii around 13–14 Å.

**Table 1:** Interaction Energy Components in kcal/mol Computed at the M062X/cc-pVDZ Level for a QM Region Containing 240 Water Molecules (*E*^240^) with MM Electrostatic Embedding Computed with the TIP3P FF

	ELE	PAU	POL	TOT	DISP	IND
ammonium	–178.1	50.9	–46.8	–173.8	–41.6	–5.2
glycine	–168.3	157.0	–108.2	–119.5	–69.6	–38.6
formate	–173.4	106.6	–64.7	–131.3	–51.2	–13.5

Ammonium and glycine show full convergence within the ±1 kcal/mol range for a QM sphere containing 160 and 70 QM water molecules (corresponding to a maximum distance of 11.3 and 9.3 Å between the atoms of the QM water molecules and solute’s center of mass), respectively, for all interaction energy components under the QM/MM-EDA approach (Figure 1a,c). On the contrary, no convergence is found for formate (Figure 1e) or for any of the solutes when full QM calculations, i.e., in the absence of the electrostatic embedding, are performed. As expected, the electrostatic component is the most difficult to converge and given its magnitude with respect to the remaining components, the total interaction energy follows a similar trend. In agreement with this observation, the zwitterionic form of glycine requires the smallest QM region to obtain converged energies.

A left to right comparison in Figure 1 reveals how the trend described by the electrostatic component and, subsequently, the total interaction energy, suffers from the absence of an electrostatic embedding. For instance, with 10 QM water molecules surrounding each solute, deviations in the total interaction energy with respect to *E*_*TOT*,*QM*/*MM*_^240^ (*E*_*TOT*,*QM*/*MM*_^240^ – *E*_*TOT*,*QM*/*MM*_^10^) of −4.4 kcal/mol, −7.3 kcal/mol, and −3.4 kcal/mol are found for ammonium, glycine and formate, respectively. By removing the MM region surrounding these QM water molecules, the energy deviations with respect to the respective reference value *E*_*TOT*,*QM*_^240^ (*E*_*TOT*,*QM*_^240^ – *E*_*TOT*,*QM*_^10^) are greatly increased to −60.8 kcal/mol, −52.1 kcal/mol, and −59.2 kcal/mol for each solute. The Pauli and dispersion components show the best convergence trends within the studied range as their short-ranged nature suggests. Indeed, within the QM/MM approach, it only takes 10, 30, and 20 QM water molecules to converge both components for the cationic, zwitterionic and anionic solutes, respectively. Larger QM regions are required to obtain similar results under a full QM scheme (Figure 1). The induction component deviates far more than dispersion for all solutes and accordingly does the polarization term. The slow convergence of the induction term is somewhat in between that of the short-range components and that of the long-range electrostatic component.

Overall, it can be seen that the introduction of a MM electrostatic embedding improves the convergence of the interaction energy and its components when comparing with the setup considering only the QM region. Nonetheless, a significant amount of water molecules is still needed to be treated quantum-mechanically for the total interaction energy to converge at reasonable accuracy, especially for systems where the long-range electrostatic interactions are likely to predominate.

**Figure 2:**
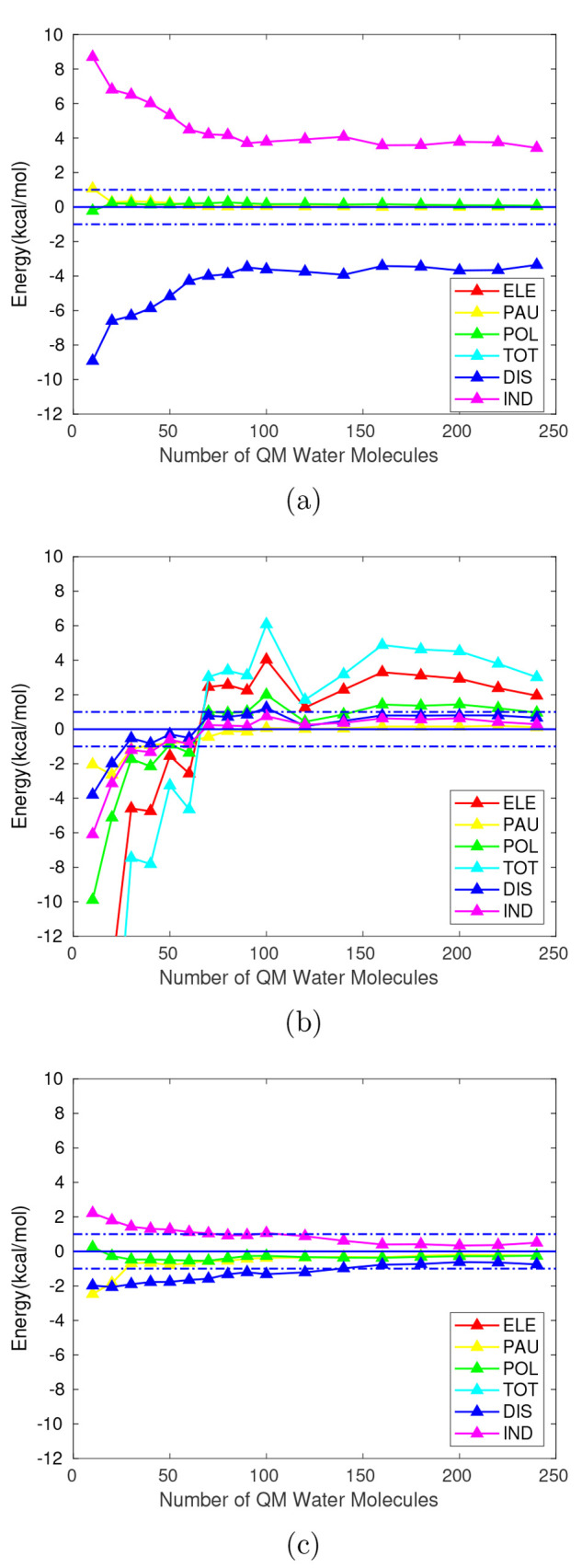
Deviation for each QM/MM interaction energy component in kcal/mol with respect to the QM energy for QM regions of equal size (*E*_*i*_^*j*,*QM*/*MM*^ – *E*_*i*_^*j*,*QM*^) for the cationic (a), zwitterionic (b), and anionic (c) solutes. Here *i* represents each component of the interaction energy, and *j* = {10, 20, ..., 240}.Total and electrostatic components are not represented for both ions for scale reasons. Horizontal dashed lines: deviation of ±1 kcal/mol.

Comparing the results obtained by the QM and the QM/MM methods, no agreement within the studied range of QM region sizes (Figure 2) is achieved. Indeed, the differences in the total and electrostatic interaction energies are quite large and, thus, they are not represented in the figures for the ionic species for scale reasons (for full scale representations check Figure S2 in the Supporting Information). The reason for this large disagreement between the QM and QM/MM calculations can be partially found on the presence of counterions in the case of the QM/MM calculations. Since charge neutrality in the simulation box is required to compute the electrostatic interactions through the Ewald summation^57,58^ as the MD simulation is performed, it was necessary to add counterions for both charged solutes. Those ions are later embedded within the MM region once the QM/MM partition of the system is performed. The removal of the MM region carried out for the full QM calculations thus leads to a significant change in the electrostatic interaction. Also, it should be emphasized that these magnitudes are not converged for the ionic QM systems (Figure 1). Removing the counterions from the QM/MM systems reduces both total and electrostatic energy differences with the QM systems from −75.0 to −58.2 kcal/mol and from −72.8 to −55.9 kcal/mol, respectively for the anion with the smallest QM water shell. A similar reduction from −116.3 to −100.8 kcal/mol and from −74.9 to −59.4 kcal/mol is found for such energy components for the cation with the QM shell containing 10 water molecules.

A common feature in all solutes can be found in the Pauli repulsion as it shows good agreement between QM and QM/MM calculations even for QM regions containing only 10 to 30 water molecules. The short-range nature of this component may be the reason for the harmony between models. Concerning polarization, it can be seen that it follows a similar trend to the Pauli repulsion, with an early convergence to the ±1 kcal/mol range. However, as Figure 2, parts a and c, make clear, this convergence results from the opposite trends shown by induction and dispersion since polarization is, indeed, sum of them both. For the neutral solute (Figure 2b) there is far better agreement for the polarization components, meeting when 60 water molecules are included in the QM partition.

In the context of a QM/MM calculation, one can aim at refining the obtained results by either improving the QM or the MM parts of the system. Regarding the former, both a systematic increase of the employed basis set and/or a better description of electron correlation by means of double-hybrid functionals, or even post-HF methods would improve the wave function of the QM region. Thus, DSD-BLYP,^71^ B2GPPLYP,^72^ and B2PLYP,^73^ all of them corrected for dispersion,^74,75^ would be an excellent choice for the study of noncovalent interactions as suggested in the literature.^76^ As for the MM part of the system, the fixed-charge TIP3P model does not allow for any sort of polarization, a relevant feature in the study of intermolecular interactions. Thus, a polarizable FF would better describe the charge distribution within the MM region by means of introducing atomic induced dipoles. This 2-fold strategy is followed from here on, assessing its impact on both the previously obtained results and their convergence.

### Effect of the Basis Set

The impact of a larger basis set (cc-pVTZ) on the different interaction energy components and the converged trends with QM region size is now assessed. For this purpose, the focus will be on systems with smaller QM regions, from 10 to 100 QM water molecules, as these differ the most from the reference value and are more susceptible to any change in the QM wave function. A QM region containing 100 water molecules corresponds to quasi-spheres of radii around 10–11 Å.

The total interaction energy behaves in a different way for each solute (Table 2). The ammonium cation shows an increase of 8.6 kcal/mol unlike glycine and the formate anion where an energy decrease of −2.2 kcal/mol and −4.8 kcal/mol, respectively, is observed for a QM region containing 100 water molecules. It should be noted that all calculations performed so far have been carried out under the CPC,^48,77^ and thus, its proneness to overcorrection for the BSSE must be taken into account.^69,70,78−80^ In this sense, the increase in the total interaction energy may reflect the reduction of the BSSE with the larger basis set. On the other hand, the anionic character of formate (and to a lesser extent that of glycine) requires the use of a larger basis set for its description, hence the energy decrease for these systems when the cc-pVDZ basis is augmented to a cc-pVTZ.

**Table 2:** Interaction Energy Components in kcal/mol Computed at the M062X/cc-pVDZ and M062X/cc-pVTZ Levels for QM Regions Containing 10 to 100 Water Molecules for the Cationic, Zwitterionic, and Anionic Solutes, with MM Electrostatic Embedding Computed with the TIP3P FF

ammonium												
	M062X/cc-pVDZ						M062X/cc-pVTZ					
# H_2_O	ELE	PAU	POL	TOT	DISP	IND	ELE	PAU	POL	TOT	DISP	IND
100	–180.6	50.9	–45.2	–174.8	–41.9	–3.3	–166.3	50.5	–50.6	–166.2	–44.3	–6.2
10	–181.8	49.8	–37.5	–169.3	–41.0	3.5	–171.8	49.7	–41.9	–163.9	–43.3	1.4
20	–184.2	50.8	–40.7	–174.0	–42.3	1.5	–172.6	50.5	–45.4	–167.5	–44.7	–0.8
30	–182.9	50.7	–41.9	–174.0	–42.0	0.1	–170.5	50.4	–46.8	–166.8	–44.6	–2.3
40	–181.4	50.7	–42.7	–173.2	–41.9	–0.9	–168.4	50.4	–47.8	–165.7	–44.4	–3.4
50	–181.1	50.8	–43.5	–173.7	–41.9	–1.6	–167.9	50.5	–48.6	–165.9	–44.4	–4.2

glycine												
	M062X/cc-pVDZ						M062X/cc-pVTZ					
# H_2_O	ELE	PAU	POL	TOT	DISP	IND	ELE	PAU	POL	TOT	DISP	IND
10	–168.7	151.6	–95.1	–112.1	–64.3	–30.8	–161.9	148.9	–101.2	–114.0	–65.4	–35.8
20	–166.8	156.1	–101.7	–112.3	–68.1	–33.6	–160.0	154.0	–109.6	–115.59	–71.7	–37.8
30	–167.9	157.0	–105.3	–116.1	–69.2	–36.1	–161.0	154.9	–113.3	–119.3	–73.5	–39.8
40	–167.1	157.2	–105.7	–115.5	–69.1	–36.7	–160.0	155.0	–113.7	–118.6	–73.5	–40.2
50	–167.3	157.2	–106.2	–116.2	–69.2	–37.0	–160.0	155.0	–114.1	–119.0	–73.6	–40.5
100	–168.8	157.0	–108.0	–119.8	–69.7	–38.3	–161.0	154.9	–115.9	–121.9	–74.0	–41.8

formate												
	M062X/cc-pVDZ						M062X/cc-pVTZ					
# H_2_O	ELE	PAU	POL	TOT	DISP	IND	ELE	PAU	POL	TOT	DISP	IND
10	–179.6	105.9	–54.4	–128.0	–49.6	–4.8	–178.8	102.2	–59.2	–135.7	–55.2	–4.0
20	–177.0	106.7	–57.8	–128.0	–50.9	–6.9	–174.9	103.0	–63.0	–134.8	–57.2	–5.8
30	–178.0	106.7	–59.8	–131.0	–51.3	–8.5	–175.2	103.1	–65.0	–137.0	–57.6	–7.4
40	–178.9	106.6	–60.6	–132.8	–51.4	–9.2	–175.6	103.0	–65.9	–138.4	–57.7	–8.2
50	–177.9	106.7	–61.2	–132.3	–51.4	–9.8	–174.4	103.0	–66.6	–137.9	–57.7	–8.9
100	–175.5	106.6	–63.0	–131.8	–51.4	–11.6	–171.1	103.0	–68.6	–136.6	–57.6	–11.0

Regarding the values obtained for the interaction energy components, it can be seen that the increase of the basis set affects each of them differently. The electrostatic component shows a significant increase for all solutes, specially for the ammonium cation (Table 2), where it rises by 14.3 kcal/mol for a QM sphere of 100 water molecules when compared to the cc-pVDZ value. On the other hand, increments of 7.8 and 4.5 kcal/mol are found for glycine and formate. Polarization follows the opposite trend, with energy decreases with respect to the computed cc-pVDZ values of −5.4 kcal/mol, −7.8 kcal/mol, and −5.6 kcal/mol for the ammonium, glycine, and formate. As expected, this trend is also followed by the two components of the polarization energy, induction and dispersion, both showing a general decrease except for the induction component in formate, where a slight increase of 0.7 kcal/mol is found. As for the Pauli repulsion, there is no significant variation when comparing results from different basis sets for ammonium, whereas for glycine and formate this component decreases with the larger basis set by −2.1 kcal/mol and −3.7 kcal/mol, respectively.

**Figure 3:**
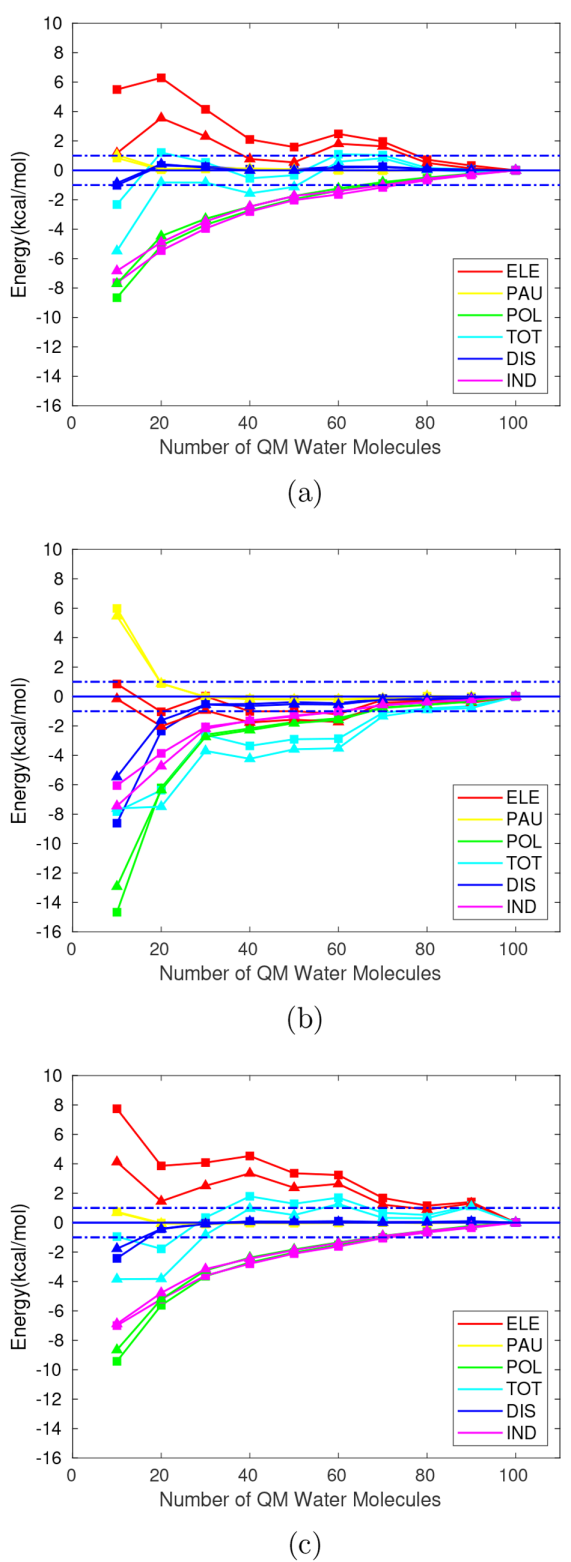
Interaction energy components in kcal/mol relative to the limit value of *E*_*i*_^100,*QMMM*^ for different sizes of the QM region within a MM (TIP3P) electrostatic embedding. Where *i* represents each interaction energy component. Ammonium (a), glycine (b), and formate (c). Squares: cc-pVTZ energies. Triangles: cc-pVDZ energies. Horizontal dashed lines: deviation of ±1 kcal/mol.

The convergence of each energy component computed with the larger cc-pVTZ basis set is compared in Figure 3 with the previous results obtained with the cc-pVDZ basis. The MM-embedded QM sphere with 100 water molecules is now considered as the reference value as we aim at comparing the convergence between both basis, not determining the convergence point. In fact, according to Figure 1, only for glycine do all the interaction energy components converge in the ±1 kcal/mol range with less than 100 waters molecules in the QM region.

From Figure 3, an improvement in the convergence of the total interaction energy when using the cc-pVTZ basis can be observed, except for glycine, where no significant change is noticed. As for the smallest QM region (containing 10 water molecules), energy deviations of −2.3 kcal/mol, −7.8 kcal/mol and −1.0 kcal/mol are found for ammonium, glycine and formate, respectively, as opposed to −5.5 kcal/mol, −7.6 kcal/mol and −3.8 kcal/mol when using the cc-pVDZ basis. Nonetheless, this improvement is not followed by the different components as their differences with respect to the reference value are increased to a greater or lesser extent when compared to the cc-pVDZ results. Hence, a larger basis set does not seem to result in an improvement of the convergence of the energy components. It is important to note that all trends represented so far correspond to a single randomly selected MD snapshot and, as such, the general tendencies here observed may vary when considering a sufficiently large sample of MD snapshots to ensure representability.

Regarding the absolute values for each component, there is a qualitative improvement when increasing the basis set. Focusing on the induction energy in Table 2, positive values appear for QM spheres from 10 to 30 QM water molecules when the cc-pVDZ basis set is considered for the ammonium cation. A positive value in the induction energy may reflect a limitation of the EDA scheme. Since the partition of polarization energy is based on second-order perturbation theory, this energy term can be exactly decomposed into induction and dispersion for weak interactions where second-order is sufficient to obtain a good approximation to the correct interaction energy. However, the intermolecular interactions between ions and polar solvents are strong and higher order terms may contribute significantly, so the pure partition of polarization into induction and dispersion may lead to unexpected results. In any case, the separate analysis of the convergence of these energy terms with the QM region size still provides useful information about the quality of the QM/MM scheme as they depend in different ways on the polarization density and on the intramolecular and intermolecular parts of the Hamiltonian. Additionally, these positive induction energies may stem also from a poor description of the potential created by the MM region or by the QM wave function. The former is addressed in the next subsection by including polarization into the MM region. The latter is partially improved when using the cc-pVTZ basis set, as can be seen in Table 2. The positive values for induction become negative for the QM region sizes of 30 and 20 water molecules and decrease for the smallest QM region of 10 water molecules.

### Polarizable Force Field

**Figure 4:**
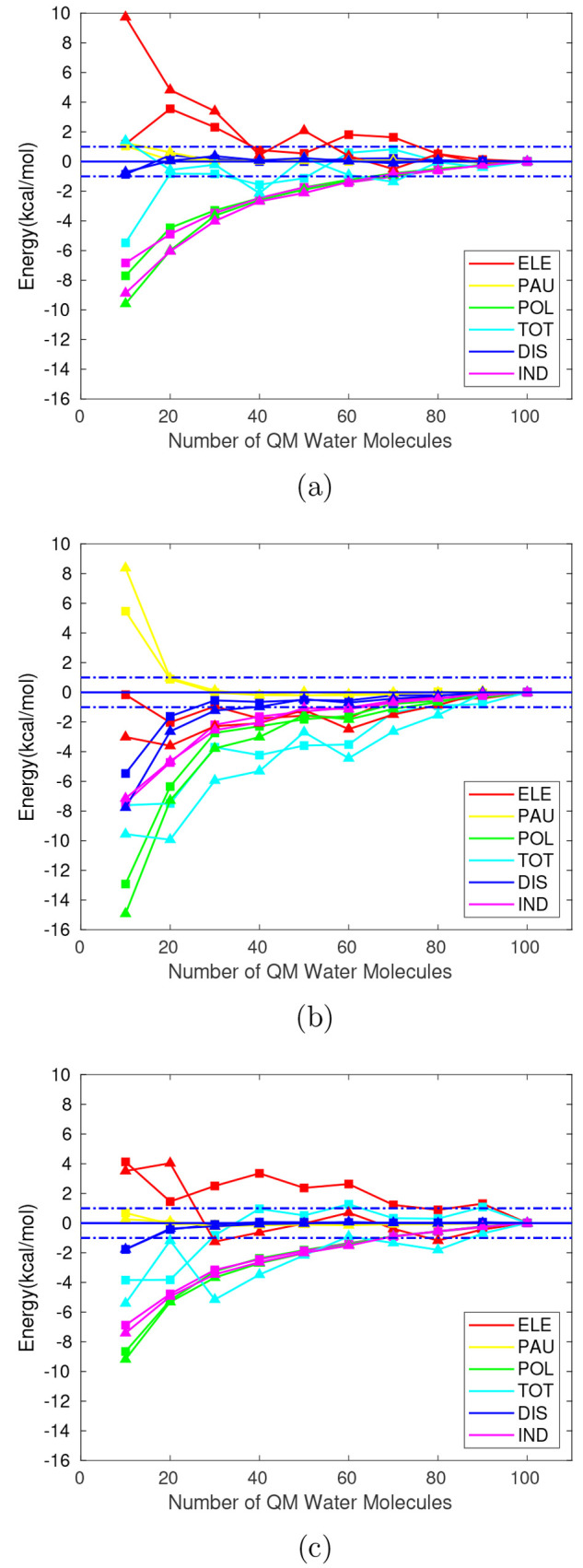
Interaction energy components in kcal/mol relative to the limit value of *E*_*i*_^100,*QMMM*^ for different sizes of the QM region within a TIP3P (squares) and POL3 (triangles) embedding. Where *i* represents each interaction energy component. Ammonium (a), glycine (b), and formate (c). Horizontal dashed lines: deviation of ±1 kcal/mol.

**Figure 5:**
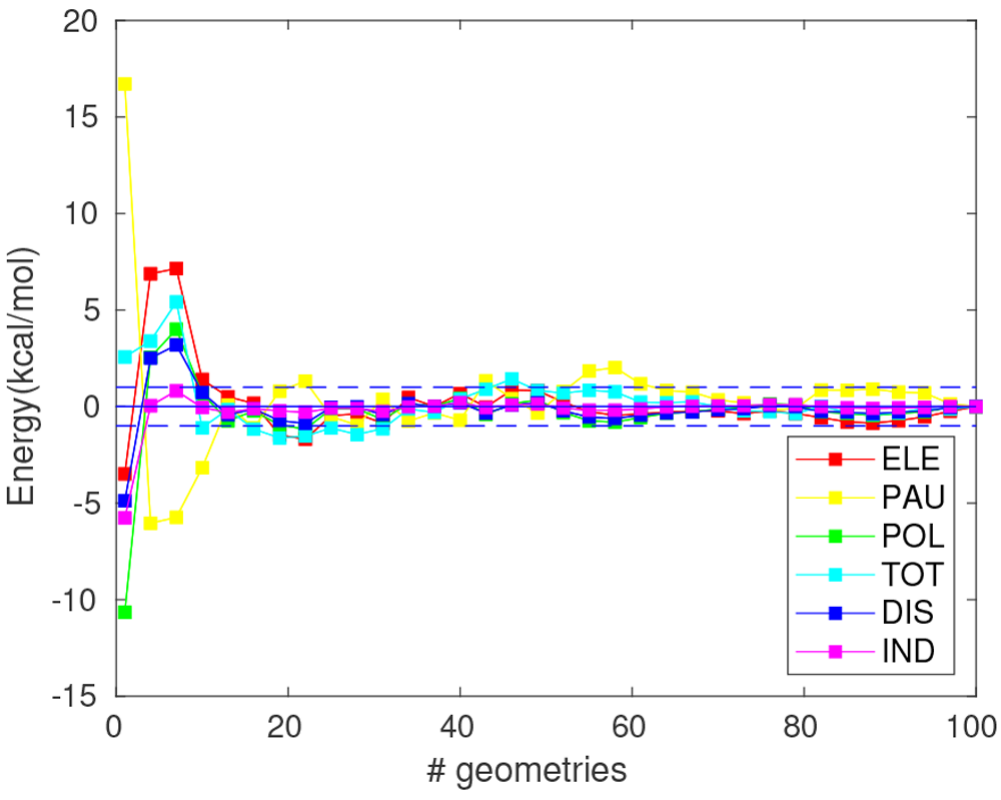
Averaged energy components in kcal/mol relative to their respective averaged value for a sample of 100 geometries with respect to the number of (equispaced) geometries in the sample. Horizontal dashed lines: deviation of ±1 kcal/mol.

**Figure 6:**
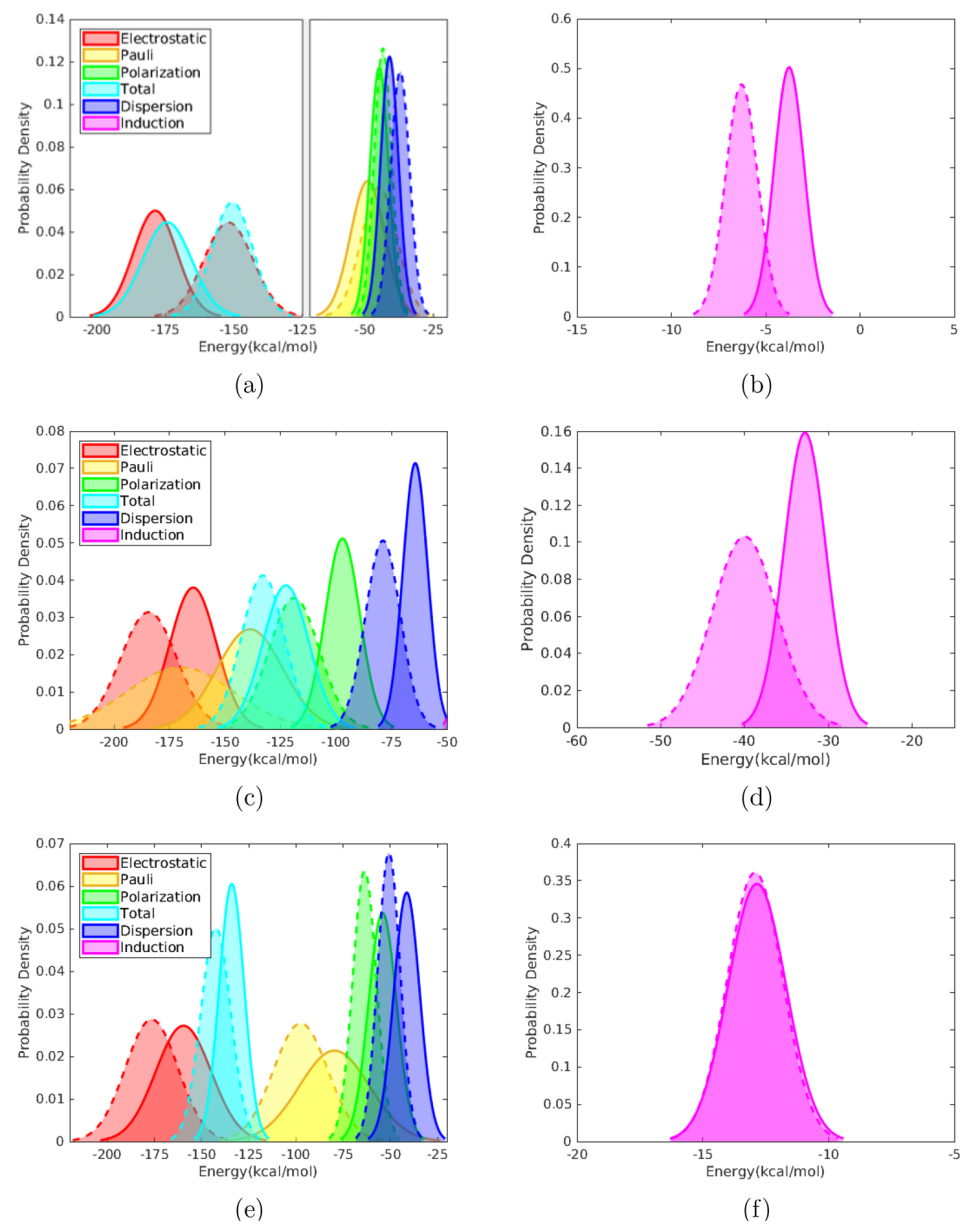
Normal probability distributions for each interaction energy component in kcal/mol obtained from a sample of 20 geometries from a MD trajectory performed with the TIP3P FF (continuous line) and the POL3 FF (dashed line). Pauli’s repulsion is represented with opposite sign. Ammonium (a and b), glycine (c and d), and formate (e and f). A vertical cut is introduced in Figure 6a to improve appearance.

At this point, the effect of including induced atomic dipoles both for the MD simulations and the EDA will be evaluated. Herein, the pure electrostatic TIP3P FF is replaced by the polarizable POL3 FF. The procedure described previously is now followed, with a focus on QM regions containing 10 to 100 water molecules for the assessment of energy convergence, obtaining the representations shown in Figure 4. It can be inferred from it how the use of the POL3 FF does not produce a general improvement in the convergence of the interaction energy components, the electrostatic energy in particular. This component converges slightly better in ionic systems, but slightly worse for zwitterionic glycine (Figure 4). In order to introduce vibration sampling in the model and compare the energy components obtained with both FFs in a more rigorous way, a first study on the convergence of the sample means with respect to the number of sampled geometries was carried out for glycine solvated by a QM water sphere containing 100 molecules. Results are summarized in Figure 5, where a sample containing 20 or more geometries was found to be sufficiently converged, as fluctuations of the sample means are in the ±1 kcal/mol range with respect to the reference when 20 or more geometries are sampled. Thus, Figure 6 represents the normal probability distributions obtained with a sample of 20 geometries from each MD trajectory considering a QM region containing 100 water molecules.

It can be observed that the distribution functions obtained for the different energy terms show a displacement toward higher or lower values depending on the studied system. Sample means (Table 3) from distributions obtained with the POL3 FF take lower values for all components in glycine; the same happens for the formate anion to a lesser extent, whereas the ammonium cation shows the opposite behavior. An exception to this behavior is found for induction in the ammonium cation where a shift toward lower values arises when using the polarizable FF, leading to an improvement with respect to the TIP3P FF results for this component, where positive values where observed for the smaller QM regions.

**Table 3:** Sample Mean (*X̅*), Standard Deviation (σ) in kcal/mol, and Relative Standard Deviation (% RSTD) for a Sample of 20 Geometries for the Cationic, Zwitterionic, and Anionic Solutes Obtained from a Classical MD Simulation with TIP3P and POL3 FFs

ammonium												
	M062X/cc-pVDZ - TIP3P						M062X/cc-pVDZ - POL3					
	ELE	PAU	POL	TOT	DISP	IND	ELE	PAU	POL	TOT	DISP	IND
*X̅*	–178.8	48.9	–44.7	–174.4	–40.9	–3.8	–152.0	44.6	–43.3	–150.6	–37.0	–6.3
σ	8.0	6.2	3.4	9.0	3.3	0.8	10.5	14.9	7.8	10.3	5.6	2.5
RSTD	4.5	12.8	7.6	5.1	8.0	21.1	5.9	14.7	7.3	4.9	9.3	13.6

glycine												
	M062X/cc-pVDZ - TIP3P						M062X/cc-pVDZ - POL3					
	ELE	PAU	POL	TOT	DISP	IND	ELE	PAU	POL	TOT	DISP	IND
*X̅*	–164.3	138.7	–97.0	–122.5	–64.2	–32.8	–184.4	170.2	–118.8	–132.9	–78.8	–40.1
σ	10.5	14.9	7.8	10.3	5.6	2.5	12.7	24.0	11.3	9.7	7.9	3.9
RSTD	6.4	10.7	8.0	8.4	8.7	7.6	6.9	14.1	9.5	7.3	10.0	9.7

formate												
	M062X/cc-pVDZ - TIP3P						M062X/cc-pVDZ - POL3					
	ELE	PAU	POL	TOT	DISP	IND	ELE	PAU	POL	TOT	DISP	IND
*X̅*	–159.7	79.6	–54.1	–134.1	–41.3	–12.8	–176.5	97.5	–63.7	–142.5	–50.7	–12.9
σ	14.7	18.7	7.4	6.6	6.8	1.2	13.9	14.4	6.3	7.9	5.9	1.1
RSTD	9.2	23.5	13.7	4.9	16.5	9.0	7.9	14.8	9.9	5.6	11.6	8.6

For all three solutes, the electrostatic component is the most important, followed by the Pauli energy, whereas dispersion and induction are the least relevant terms with values that vary greatly from system to system. In fact, it can be appreciated how the electrostatic component accounts for most of the interaction energy in ionic solutes, with the polarization components playing less of a role, especially for the ammonium cation, in agreement with its less polarizable character. This is no longer the case for glycine, where it can be seen how polarization contributes significantly to the interaction energy.

Regarding the width of the distribution functions (Figure 6), it is worth mentioning the qualitative correlation between the relative standard deviation (RSTD) and the short or long-range character of each component. There is a direct relation between the average value of each distribution and its STD, however, when calculating the respective RSTDs, it can be seen how the short-range Pauli repulsion corresponds to the largest values, whereas the long-range electrostatic or the total interaction energy show the smallest ones as it can be inferred from Table 3. From the relatively large STDs, especially for short-range interactions, the importance of the configurational sampling, in this case performed with classical MD, is restated for the analysis of solute–solvent interactions. Interestingly, the use of a polarizable FF results in a broadening of the distributions for ammonium and particularly for glycine and its Pauli term, whereas the opposite is true for the formate anion.

Focusing now on the averaged values of the interaction energy components, it can be seen from both Figure 6 and Table 3 that there is no clear pattern regarding the distributions obtained with different FFs. For glycine and the formate anion, a displacement toward lower values appears when using the polarizable FF, whereas the opposite is true for the ammonium cation. The limitations when implementing polarizable FFs for the EDA-QM/MM analysis are reflected on the induction component, where negligible differences in its distributions are observed for the formate anion, whereas a displacement toward lower values appears for ammonium and glycine. The QM/MM-EDA scheme employed here, where the induced atomic dipoles of the MM solvent region are directly taken from the MD simulation of the whole system, does not allow to include in the interaction energy the effect of the changes in the induced atomic dipoles of the MM region due to the solute, an effect that it is certainly at the heart of the induction energy. Thus, it is not an unexpected finding that the induction energy values barely change from the pure electrostatic to the polarizable FF when the QM region is large (100 water molecules) for the anionic solute.

## Conclusions

In this work, the solute–solvent interaction energy components of a series of ionic solutes in water have been investigated under our proposed QM/MM-EDA scheme in order to understand how the setup of the QM/MM calculation affects the convergence of such energy components with the QM region size.

By means of a classical MD trajectory, a series of conformations have been obtained for each solute in water solution, selecting one for the convergence study with QM region size at the M062X/cc-pVDZ level. A range of 10–240 QM water molecules has been included and a strong improvement has been found in the overall trends when the MM embedding is explicitly introduced in the EDA scheme (QM/MM-EDA). A reduction from −59.2 to −3.4 kcal/mol has been found in the interaction energy difference between a QM region containing 10 and 240 water molecules when going from a fully QM to a QM/MM-EDA scheme in the case of the formate anion.

The convergence is strongly dependent on the electrostatic energy component and, thus, a significantly large number of explicit water molecules must be treated quantum-mechanically to converge all components at chemical accuracy. Indeed, as many as 160 and 70 water molecules must be included in the QM region to reach convergence for all components at the ±1 kcal/mol range for ammonium and glycine, respectively. As numerically shown above, no convergence has been found with the chosen criteria for formate anion. Hence, it has been shown that, for the studied solute–solvent interactions, including a nonnegligible amount of solvent molecules in the QM region becomes necessary, and thus defining the QM region with just the solute and the first solvation sphere is a rough approximation in QM/MM calculations.

Using a larger cc-pVTZ basis set does improve the convergence of the total interaction energy but not that of different interaction energy components. The augmentation of the basis set yields a general increase of the electrostatic energy, whereas the polarization energy decreases and Pauli slightly changes. This result is in agreement with the importance of using large basis sets to accurately represent the molecular electric polarizability. In addition, the use of a large basis set partially corrects the ill behavior of having positive values for the induction component when small QM regions are employed.

When introducing induced atomic dipoles in the MM region by means of the POL3 FF, no clear trend has been found by comparing the normal distributions of each energy component with those obtained with the electrostatic embedding based on atomic charges, as displacements toward higher and lower values appear depending on the nature of the solute. However, a decrease in induction for the ammonium cation is observed, improving previous results from the TIP3P atomic charges. The width of the obtained distributions supports the need for configurational sampling, especially for the short-range Pauli component. Regarding convergence, no significant improvement has been found when introducing induced atomic dipoles in the electrostatic embedding.

## Data Availability

The tleap and antechamber packages from the AmberTools20^50^ toolkit were used to generate the topology and the coordinate files for the MD simulations. The PMEMD.CUDA^54,55^ and SANDER modules of the AMBER20^50^ (https://ambermd.org/) software were used to perform the classical MD simulations. The MoBioTools toolkit (https://github.com/mobiochem/MoBioTools) was used to automatically generate the QM/MM input files. QM/MM computations themselves were performed with the Gaussian16^61^ (https://gaussian.com/) package. The EDA-NCI program (https://github.com/marcos-mandado/EDA-NCI) was used to perform the EDA. Trajectories were visualized with the Visual Molecular Dynamics (VMD, https://www.ks.uiuc.edu/Research/vmd/).

## Supporting Information Available

Figures S1 and S2, corresponding to the full scale representations of Figures 1 and 2 in the main text, respectively(PDF)

## References

[ref1] Mansoor E.; Van der Mynsbrugge J.; Head-Gordon M.; Bell A. T. Impact of long-range electrostatic and dispersive interactions on theoretical predictions of adsorption and catalysis in zeolites. Catal. Today 2018, 312, 51–65. 10.1016/j.cattod.2018.02.007.

[ref2] Norberg J.; Nilsson L. On the truncation of long-range electrostatic interactions in DNA. Biophys. J. 2000, 79, 1537–1553. 10.1016/S0006-3495(00)76405-8. 10969015 PMC1301047

[ref3] Pabbathi A.; Coleman L.; Godar S.; Paul A.; Garlapati A.; Spencer M.; Eller J.; Alper J. D. Long-range electrostatic interactions significantly modulate the affinity of dynein for microtubules. Biophys. J. 2022, 121, 1715–1726. 10.1016/j.bpj.2022.03.029. 35346642 PMC9117880

[ref4] Chari R.; Jerath K.; Badkar A. V.; Kalonia D. S. Long- and short-range electrostatic interactions affect the rheology of highly concentrated antibody solutions. Pharm. Res. 2009, 26, 2607–2618. 10.1007/s11095-009-9975-2. 19795191

[ref5] Yue S.; Muniz M. C.; Calegari Andrade M. F.; Zhang L.; Car R.; Panagiotopoulos A. Z. When do short-range atomistic machine-learning models fall short. J. Chem. Phys. 2021, 154, 034111 10.1063/5.0031215. 33499637

[ref6] Senn H. M.; Thiel W. QM/MM methods for biomolecular systems. Angew. Chem., Int. Ed. 2009, 48, 1198–1229. 10.1002/anie.200802019. 19173328

[ref7] Nogueira J. J.; González L. Computational Photophysics in the Presence of an Environment. Annu. Rev. Phys. Chem. 2018, 69, 473–497. 10.1146/annurev-physchem-050317-021013. 29490201

[ref8] Brunk E.; Rothlisberger U. Mixed quantum mechanical/molecular mechanical molecular dynamics simulations of biological systems in ground and electronically excited states. Chem. Rev. 2015, 115, 6217–6263. 10.1021/cr500628b. 25880693

[ref9] Olsen J. M. H.; Steinmann C.; Ruud K.; Kongsted J. Polarizable density embedding: A new QM/QM/MM-based computational strategy. J. Phys. Chem. A 2015, 119, 5344–5355. 10.1021/jp510138k. 25594604

[ref10] Wesolowski T. A.; Shedge S.; Zhou X. Frozen-Density Embedding Strategy for Multilevel Simulations of Electronic Structure. Chem. Rev. 2015, 115, 5891–5928. 10.1021/cr500502v. 25923542

[ref11] Nogueira J. J.; González L. Computational Photophysics in the Presence of an Environment. Annu. Rev. Phys. Chem. 2018, 69, 473–497. 10.1146/annurev-physchem-050317-021013. 29490201

[ref12] Cao L.; Ryde U. On the difference between additive and subtractive QM/MM calculations. Front. Chem. 2018, 6, 00089 10.3389/fchem.2018.00089. PMC5891596 29666794

[ref13] Sherwood P.; De Vries A. H.; Guest M. F.; Schreckenbach G.; Catlow C. R. A.; French S. A.; Sokol A. A.; Bromley S. T.; Thiel W.; Turner A. J.; Billeter S.; Terstegen F.; Thiel S.; Kendrick J.; Rogers S. C.; Casci J.; Watson M.; King F.; Karlsen E.; Sjøvoll M.; Fahmi A.; Schäfer A.; Lennartz C. QUASI: A general purpose implementation of the QM/MM approach and its application to problems in catalysis. J. Mol. Struct.: THEOCHEM 2003, 632, 1–28. 10.1016/S0166-1280(03)00285-9.

[ref14] Senn H. M.; Thiel W. QM/MM methods for biomolecular systems. Angew. Chem., Int. Ed. 2009, 48, 1198–1229. 10.1002/anie.200802019. 19173328

[ref15] Maseras F.; Morokuma K. IMOMM: A new integrated ab initio + molecular mechanics geometry optimization scheme of equilibrium structures and transition states. J. Comput. Chem. 1995, 16, 1170–1179. 10.1002/jcc.540160911.

[ref16] Svensson M.; Humbel S.; Froese R. D. J.; Matsubara T.; Sieber S.; Morokuma K. ONIOM: A multilayered integrated MO + MM method for geometry optimizations and single point energy predictions. A test for Diels-Alder reactions and Pt(P(t-Bu)3)2 + H2 oxidative addition. J. Phys. Chem. 1996, 100, 19357–19363. 10.1021/jp962071j.

[ref17] Chung L. W.; Sameera W. M. C.; Ramozzi R.; Page A. J.; Hatanaka M.; Petrova G. P.; Harris T. V.; Li X.; Ke Z.; Liu F.; Li H.; Ding L.; Morokuma K. The ONIOM method and its applications. Chem. Rev. 2015, 115, 5678–5796. 10.1021/cr5004419. 25853797

[ref18] Svensson M.; Humbel S.; Froese R. D. J.; Matsubara T.; Sieber S.; Morokuma K. ONIOM: A multilayered integrated MO + MM method for geometry optimizations and single point energy predictions. A test for Diels-Alder reactions and Pt(P(t-Bu)3)2 + H2 oxidative addition. J. Phys. Chem. 1996, 100, 19357 10.1021/jp962071j.

[ref19] Field M. J.; Bash P. A.; Karplus M. A combined quantum mechanical and molecular mechanical potential for molecular dynamics simulations. J. Comput. Chem. 1990, 11, 700–733. 10.1002/jcc.540110605.

[ref20] Ryde U. The coordination of the catalytic zinc ion in alcohol dehydrogenase studied by combined quantum-chemical and molecular mechanics calculations. J. Comput.-Aided Mol. Des. 1996, 10, 153–164. 10.1007/BF00402823. 8741019

[ref21] Dapprich S.; Komáromi I.; Byun K. S.; Morokuma K.; Frisch M. J. A new ONIOM implementation in Gaussian98. Part I. The calculation of energies, gradients, vibrational frequencies and electric field derivatives. J. Mol. Struct.: THEOCHEM 1999, 461–462, 1–21. 10.1016/S0166-1280(98)00475-8.

[ref22] Poulsen T. D.; Kongsted J.; Osted A.; Ogilby P. R.; Mikkelsen K. V. The combined multiconfigurational self-consistent-field/molecular mechanics wave function approach. J. Chem. Phys. 2001, 115, 2393–2400. 10.1063/1.1374559.

[ref23] Söderhjelm P.; Husberg C.; Strambi A.; Olivucci M.; Ryde U. Protein influence on electronic spectra modeled by multipoles and polarizabilities. J. Chem. Theory Comput. 2009, 5, 649–658. 10.1021/ct800459t. 26610229

[ref24] Olsen J. M.; Aidas K.; Kongsted J. Excited states in solution through polarizable embedding. J. Chem. Theory Comput. 2010, 6, 3721–3734. 10.1021/ct1003803.

[ref25] Zhang Y.; Lin H. Flexible-boundary QM/MM calculations: II. partial charge transfer across the QM/MM boundary that passes through a covalent bond. Theor. Chem. Acc. 2010, 126, 315–322. 10.1007/s00214-009-0704-z.

[ref26] Zhang Y.; Lin H. Flexidle-boundry quantum-mechanical/molecular-mechanical calculations: partial charge transfer between the quantum-mechanical and molecular-mechanical subsystems. J. Chem. Theory Comput. 2008, 4, 414–425. 10.1021/ct700296x. 26620782

[ref27] Milanese J. M.; Provorse M. R.; Alameda J. E.; Isborn C. M. Convergence of computed aqueous absorption spectra with explicit quantum mechanical solvent. J. Chem. Theory Comput. 2017, 13, 2159–2171. 10.1021/acs.jctc.7b00159. 28362490

[ref28] Isborn C. M.; Götz A. W.; Clark M. A.; Walker R. C.; Martínez T. J. Electronic absorption spectra from MM and ab initio QM/MM molecular dynamics: environmental effects on the absorption spectrum of photoactive yellow protein. J. Chem. Theory Comput. 2012, 8, 5092–5106. 10.1021/ct3006826. 23476156 PMC3590007

[ref29] Nogueira J. J.; Plasser F.; González L. Electronic delocalization, charge transfer and hypochromism in the UV absorption spectrum of polyadenine unravelled by multiscale computations and quantitative wavefunction analysis. Chem. Sci. 2017, 8, 5682–5691. 10.1039/C7SC01600J. 28989607 PMC5621053

[ref30] Ibele L. M.; Sánchez-Murcia P. A.; Mai S.; Nogueira J. J.; González L. Excimer intermediates en route to long-lived charge-transfer states in single-stranded adenine DNA as revealed by nonadiabatic dynamics. J. Phys. Chem. Lett. 2020, 11, 7483–7488. 10.1021/acs.jpclett.0c02193. 32794719 PMC7503858

[ref31] Provorse M. R.; Peev T.; Xiong C.; Isborn C. M. Convergence of excitation energies in mixed quantum and classical solvent: comparison of continuum and point charge models. J. Phys. Chem. B 2016, 120, 12148–12159. 10.1021/acs.jpcb.6b09176. 27797196

[ref32] Isborn C. M.; Mar B. D.; Curchod B. F. E.; Tavernelli I.; Martínez T. J. The charge transfer problem in density functional theory calculations of aqueously solvated molecules. J. Phys. Chem. B 2013, 117, 12189–12201. 10.1021/jp4058274. 23964865

[ref33] Flaig D.; Beer M.; Ochsenfeld C. Convergence of electronic structure with the size of the QM region: Example of QM/MM NMR shieldings. J. Chem. Theory Comput. 2012, 8, 2260–2271. 10.1021/ct300036s. 26588959

[ref34] Roßbach S.; Ochsenfeld C. Influence of coupling and embedding schemes on QM size convergence in QM/MM approaches for the example of a proton transfer in DNA. J. Chem. Theory Comput. 2017, 13, 1102–1107. 10.1021/acs.jctc.6b00727. 28195707

[ref35] Das S.; Nam K.; Major D. T. Rapid convergence of energy and free energy profiles with quantum mechanical size in quantum mechanical-molecular mechanical simulations of proton transfer in DNA. J. Chem. Theory Comput. 2018, 14, 1695–1705. 10.1021/acs.jctc.7b00964. 29446946

[ref36] Jindal G.; Warshel A. Exploring the dependence of QM/MM calculations of enzyme catalysis on the size of the QM region. J. Phys. Chem. B 2016, 120, 9913–9921. 10.1021/acs.jpcb.6b07203. 27552257 PMC5036132

[ref37] Liao R.; Thiel W. Convergence in the QM-only and QM/MM modeling of enzymatic reactions: A case study for acetylene hydratase. J. Comput. Chem. 2013, 34, 2389–2397. 10.1002/jcc.23403. 23913757

[ref38] Kulik H. J.; Zhang J.; Klinman J. P.; Martínez T. J. How large should the QM region be in QM/MM calculations? the case of catechol O-methyltransferase. J. Phys. Chem. B 2016, 120, 11381–11394. 10.1021/acs.jpcb.6b07814. 27704827 PMC5108028

[ref39] Karelina M.; Kulik H. J. Systematic quantum mechanical region determination in QM/MM simulation. J. Chem. Theory Comput. 2017, 13, 563–576. 10.1021/acs.jctc.6b01049. 28068092

[ref40] Fox S. J.; Pittock C.; Fox T.; Tautermann C. S.; Malcolm N.; Skylaris C. Electrostatic embedding in large-scale first principles quantum mechanical calculations on biomolecules. J. Chem. Phys. 2011, 135, 224107 10.1063/1.3665893. 22168680

[ref41] Mandado M.; Hermida-Ramón J. M. Electron density based partitioning scheme of interaction energies. J. Chem. Theory Comput. 2011, 7, 633–641. 10.1021/ct100730a. 26596297

[ref42] Ramos-Berdullas N.; Pérez-Juste I.; Van Alsenoy C.; Mandado M. Theoretical study of the adsorption of aromatic units on carbon allotropes including explicit (empirical) DFT dispersion corrections and implicitly dispersion-corrected functionals: The pyridine case. Phys. Chem. Chem. Phys. 2015, 17, 575–587. 10.1039/C4CP02341B. 25407229

[ref43] Cárdenas G.; Pérez-Barcia; Mandado M.; Nogueira J. J. Characterization of cisplatin/membrane interactions by QM/MM energy decomposition analysis. Phys. Chem. Chem. Phys. 2021, 23, 20533–20540. 10.1039/D1CP03382D. 34505588

[ref44] López R.; Díaz N.; Francisco E.; Martín-Pendás A.; Suárez D. QM/MM energy decomposition using the interacting quantum atoms approach. J. Chem. Inf. Model. 2022, 62, 1510–1524. 10.1021/acs.jcim.1c01372. 35212531 PMC8965874

[ref45] Mao Y.; Shao Y.; Dziedzic J.; Skylaris C.; Head-Gordon T.; Head-Gordon M. Performance of the AMOEBA water model in the vicinity of QM solutes: a diagnosis using Energy Decomposition Analysis. J. Chem. Theory Comput. 2017, 13, 1963–1979. 10.1021/acs.jctc.7b00089. 28430427

[ref46] Dziedzic J.; Head-Gordon T.; Head-Gordon M.; Skylaris C. Mutually polarizable QM/MM model with in situ optimized localized basis functions. J. Chem. Phys. 2019, 150, 074103 10.1063/1.5080384. 30795653

[ref47] Alvarado R.; Cardenas G.; Nogueira J.; Ramos-Berdullas N.; Mandado M. On the Permeation of Polychlorinated Dibenzodioxins and Dibenzofurans through Lipid Membranes: Classical MD and Hybrid QM/MM-EDA Analysis. Membranes 2023, 13, 28 10.3390/membranes13010028. PMC9865757 36676835

[ref48] Boys S. F.; Bernardi F. The calculation of small molecular interactions by the differences of separate total energies. Some procedures with reduced errors. Mol. Phys. 1970, 19, 553–566. 10.1080/00268977000101561.

[ref49] Kaplan I. G. Intermolecular Interactions: Physical Picture, Computational Methods, Model Potentials; John Wiley & Sons: Chichester, England, 2006.

[ref50] Case D.; Aktulga H.; Belfon K.; Ben-Shalom I.; Brozell S.; Cerutti D.; III T. C.; Cisneros G.; Cruzeiro V.; Darden T.; Duke R.; Giambasu G.; Gilson M.; Gohlke H.; Goetz A.; Harris R.; Izadi S.; Izmailov S.; Jin C.; Kasavajhala K.; Kaymak M.; King E.; Kovalenko A.; Kurtzman T.; Lee T.; LeGrand S.; Li P.; Lin C.; Liu J.; Luchko T.; Luo R.; Machado M.; Man V.; Manathunga M.; Merz K.; Miao Y.; Mikhailovskii O.; Monard G.; Nguyen H.; O’Hearn K.; Onufriev A.; Pan F.; Pantano S.; Qi R.; Rahnamoun A.; Roe D.; Roitberg A.; Sagui C.; Schott-Verdugo S.; Shen J.; Simmerling C.; Skrynnikov N.; Smith J.; Swails J.; Walker R.; Wang J.; Wei H.; Wolf R.; Wu X.; Xue Y.; York D.; Zhao S.; Kollman P. AMBER 2020; 2020.

[ref51] Wang J.; Wolf R. M.; Caldwell J. W.; Kollman P. A.; Case D. A. Development and testing of a general Amber force field. J. Comput. Chem. 2004, 25, 1157–1174. 10.1002/jcc.20035. 15116359

[ref52] Jorgensen W. L.; Chandrasekhar J.; Madura J. D.; Impey R. W.; Klein M. L. Comparison of simple potential functions for simulating liquid water. J. Chem. Phys. 1983, 79, 926–935. 10.1063/1.445869.

[ref53] Caldwell J. W.; Kollman P. A. Structure and properties of neat liquids using nonadditive molecular dynamics: Water, methanol, and N-methylacetamide. J. Phys. Chem. 1995, 99, 6208–6219. 10.1021/j100016a067.

[ref54] Götz A. W.; Williamson M. J.; Xu D.; Poole D.; Le Grand S.; Walker R. C. Routine Microsecond Molecular Dynamics Simulations with AMBER on GPUs. 1. Generalized Born. J. Chem. Theory Comput. 2012, 8, 1542–1555. 10.1021/ct200909j. 22582031 PMC3348677

[ref55] Salomon-Ferrer R.; Götz A. W.; Poole D.; Le Grand S.; Walker R. C. Routine Microsecond Molecular Dynamics Simulations with AMBER on GPUs. 2. Explicit Solvent Particle Mesh Ewald. J. Chem. Theory Comput. 2013, 9, 3878–3888. 10.1021/ct400314y. 26592383

[ref56] Berendsen H. J. C.; Postma J. P. M.; van Gunsteren W. F.; DiNola A.; Haak J. R. Molecular dynamics with coupling to an external bath. J. Chem. Phys. 1984, 81, 3684 10.1063/1.448118.

[ref57] Ewald P. P. Die Berechnung optischer und elektrostatischer Gitterpotentiale. Ann. Phys. 1921, 369, 253–287. 10.1002/andp.19213690304.

[ref58] Darden T.; York D.; Pedersen L. Particle mesh Ewald: An N·log(N) method for Ewald sums in large systems. J. Chem. Phys. 1993, 98, 10089–10092. 10.1063/1.464397.

[ref59] Li P.; Song L. F.; Merz K. M. Systematic parameterization of monovalent ions employing the nonbonded model. J. Chem. Theory Comput. 2015, 11, 1645–1657. 10.1021/ct500918t. 26574374

[ref60] Miyamoto S.; Kollman P. A. Settle: An analytical version of the SHAKE and RATTLE algorithm for rigid water models. J. Comput. Chem. 1992, 13, 952–962. 10.1002/jcc.540130805.

[ref61] Frisch M. J.; Trucks G. W.; Schlegel H. B.; Scuseria G. E.; Robb M. A.; Cheeseman J. R.; Scalmani G.; Barone V.; Petersson G. A.; Nakatsuji H.; Li X.; Caricato M.; Marenich A. V.; Bloino J.; Janesko B. G.; Gomperts R.; Mennucci B.; Hratchian H. P.; Ortiz J. V.; Izmaylov A. F.; Sonnenberg J. L.; Williams-Young D.; Ding F.; Lipparini F.; Egidi F.; Goings J.; Peng B.; Petrone A.; Henderson T.; Ranasinghe D.; Zakrzewski V. G.; Gao J.; Rega N.; Zheng G.; Liang W.; Hada M.; Ehara M.; Toyota K.; Fukuda R.; Hasegawa J.; Ishida M.; Nakajima T.; Honda Y.; Kitao O.; Nakai H.; Vreven T.; Throssell K.; Montgomery J. A.; Peralta J. E.; Ogliaro F.; Bearpark M. J.; Heyd J. J.; Brothers E. N.; Kudin K. N.; Staroverov V. N.; Keith T. A.; Kobayashi R.; Normand J.; Raghavachari K.; Rendell A. P.; Burant J. C.; Iyengar S. S.; Tomasi J.; Cossi M.; Millam J. M.; Klene M.; Adamo C.; Cammi R.; Ochterski J. W.; Martin R. L.; Morokuma K.; Farkas O.; Foresman J. B.; Fox D. J. Gaussian 16, Revision C.01; 2016,.

[ref62] Zhao Y.; Truhlar D. G. The M06 suite of density functionals for main group thermochemistry, thermochemical kinetics, noncovalent interactions, excited states, and transition elements: Two new functionals and systematic testing of four M06-class functionals and 12 other functionals. Theor. Chem. Acc. 2008, 120, 215–241. 10.1007/s00214-007-0310-x.

[ref63] Dunning T. H. Jr. Gaussian basis sets for use in correlated molecular calculations. I. The atoms boron through neon and hydrogen. J. Chem. Phys. 1989, 90, 1007–1023. 10.1063/1.456153.

[ref64] Kendall R. A.; Dunning T. H. Jr.; Harrison R. J. Electron affinities of the first-row atoms revisited. Systematic basis sets and wave functions. J. Chem. Phys. 1992, 96, 6796–6806. 10.1063/1.462569.

[ref65] Woon D. E.; Dunning T. H. Jr. Gaussian basis sets for use in correlated molecular calculations. III. The atoms aluminum through argon. J. Chem. Phys. 1993, 98, 1358–1371. 10.1063/1.464303.

[ref66] Peterson K. A.; Woon D. E.; Dunning T. H. Jr. Benchmark calculations with correlated molecular wave functions. IV. The classical barrier height of the H+H2→H2+H reaction. J. Chem. Phys. 1994, 100, 7410–7415. 10.1063/1.466884.

[ref67] Wilson A. K.; Van Mourik T.; Dunning T. H. Jr. Gaussian basis sets for use in correlated molecular calculations. VI. Sextuple zeta correlation consistent basis sets for boron through neon. J. Mol. Struct.: THEOCHEM 1996, 388, 339–349. 10.1016/S0166-1280(96)80048-0.

[ref68] Cárdenas G.; Lucia-Tamudo J.; Mateo-delaFuente H.; Palmisano V. F.; Anguita-Ortiz N.; Ruano L.; Pérez-Barcia A.; Díaz-Tendero S.; Mandado M.; Nogueira J. J. MoBioTools: A Toolkit to Setup QM/MM Calculations. J. Comput. Chem. 2023, 44, 516–533. 10.1002/jcc.27018. 36507763 PMC10107847

[ref69] Johansson A.; Kollman P.; Rothenberg S. An application of the functional Boys-Bernardi counterpoise method to molecular potential surfaces. Theor. Chim. Acta 1973, 29, 167–172. 10.1007/BF00529439.

[ref70] Daudey J. P.; Claverie P.; Malrieu J. P. Perturbative ab initio calculations of intermolecular energies. I. Method. Int. J. Quantum Chem. 1974, 8, 1–15. 10.1002/qua.560080102.

[ref71] Kozuch S.; Gruzman D.; Martin J. M. L. DSD-BLYP: A general purpose double hybrid density functional including spin component scaling and dispersion correction. J. Phys. Chem. C 2010, 114, 20801–20808. 10.1021/jp1070852.

[ref72] Karton A.; Tarnopolsky A.; Lamére J.; Schatz G. C.; Martin J. M. L. Highly accurate first-principles benchmark data sets for the parametrization and validation of density functional and other approximate methods. Derivation of a robust, generally applicable, double-hybrid functional for thermochemistry and thermochemical kinetics. J. Phys. Chem. A 2008, 112, 12868–12886. 10.1021/jp801805p. 18714947

[ref73] Grimme S. Semiempirical hybrid density functional with perturbative second-order correlation. J. Chem. Phys. 2006, 124, 034108 10.1063/1.2148954. 16438568

[ref74] Grimme S.; Antony J.; Ehrlich S.; Krieg H. A consistent and accurate ab initio parametrization of density functional dispersion correction (DFT-D) for the 94 elements H-Pu. J. Chem. Phys. 2010, 132, 154104 10.1063/1.3382344. 20423165

[ref75] Grimme S.; Ehrlich S.; Goerigk L. Effect of the damping function in dispersion corrected density functional theory. J. Comput. Chem. 2011, 32, 1456–1465. 10.1002/jcc.21759. 21370243

[ref76] Goerigk L.; Hansen A.; Bauer C.; Ehrlich S.; Najibi A.; Grimme S. A look at the density functional theory zoo with the advanced GMTKN55 database for general main group thermochemistry, kinetics and noncovalent interactions. Phys. Chem. Chem. Phys. 2017, 19, 32184–32215. 10.1039/C7CP04913G. 29110012

[ref77] Meunier A.; Lévy B.; Berthier G. Electron correlation and basis effects in the theory of hydrogen bonds: The mixed dimer ammonia-water. Theor. Chim. Acta 1973, 29, 49–55. 10.1007/BF00528166.

[ref78] Frisch M. J.; Del Bene J. E.; Binkley J. S.; Schaefer H. F. III Extensive theoretical studies of the hydrogen-bonded complexes (H 2O)2, (H2O)2H+, (HF) 2, (HF)2H+, F2H+, and (NH3)2. J. Chem. Phys. 1986, 84, 2279–2289. 10.1063/1.450390.

[ref79] López J. C.; Alonso J. L.; Lorenzo F. J.; Rayón V. M.; Sordo J. A. The tetrahydrofuranhydrogen chloride complex: Rotational spectrum and theoretical analysis. J. Chem. Phys. 1999, 111, 6363–6374. 10.1063/1.479962.

[ref80] Valdés H.; Sordo J. A. Ab initio study on the (OCS)2·CO2 van der Waals trimers. J. Phys. Chem. A 2002, 106, 3690–3701. 10.1021/jp0128168. 11908080

