# Supplementary material for: Effect of the QM Size, Basis Set, and Polarization on QM/MM Interaction Energy Decomposition Analysis
Source: J Chem Inf Model. 2023 Jan 20;63(3):882–97. doi: 10.1021/acs.jcim.2c01184 (PMC9930123; doi:10.1021/acs.jcim.2c01184)
Supplement: Supplementary file 1 — ci2c01184_si_002.pdf [file ci2c01184_si_002.pdf]

Supporting Information for "Effect of the QM Size, Basis Set and Polarization on QM/MM Interaction Energy Decomposition Analysis"

Álvaro Pérez-Barcia,† Gustavo Cárdenas,‡ Juan J. Nogueira,*,‡ and Marcos Mandado*,†,¶

†*Department of Physical Chemistry, University of Vigo, Lagoas-Marcosende s\n, ES-36310-Vigo, Galicia, Spain*

‡*Department of Chemistry, Universidad Autónoma de Madrid, Calle Francisco Tomás y Valiente, 7, 28049, Madrid, Spain*

¶*IADCHEM, Institute for Advanced Research in Chemistry, Universidad Autónoma de Madrid, Calle Francisco Tomás y Valiente, 7, 28049 Madrid, Spain*

E-mail: juan.nogueira@uam.es; mandado@uvigo.es

This section contains the full scale figures corresponding to Figures 1 and 2 in the main text.

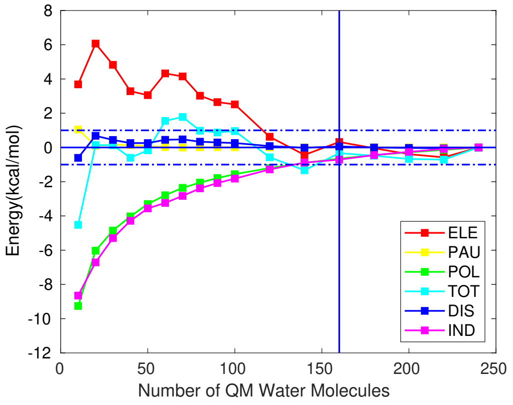

(a)

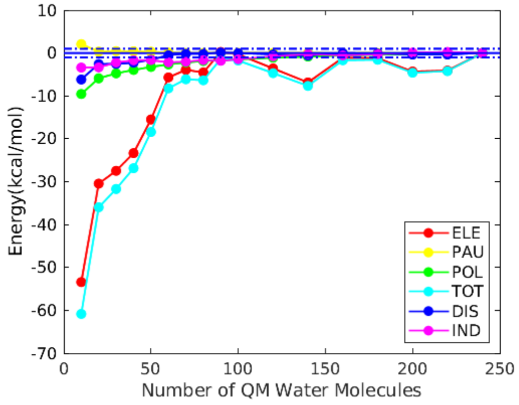

(b)

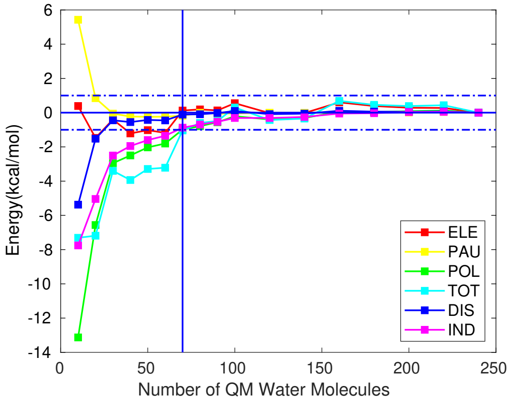

(c)

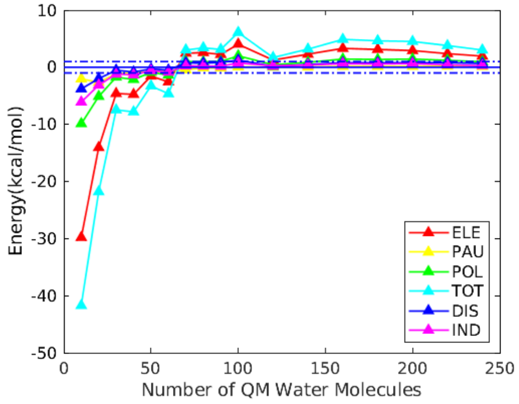

(d)

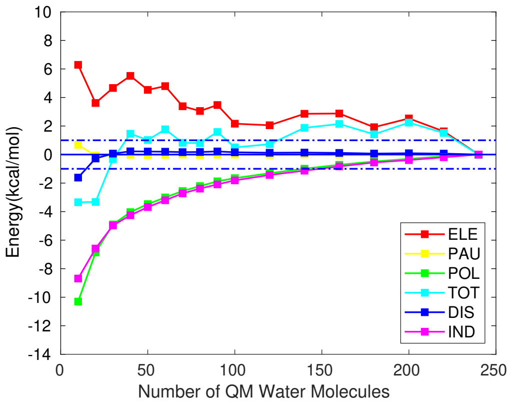

(e)

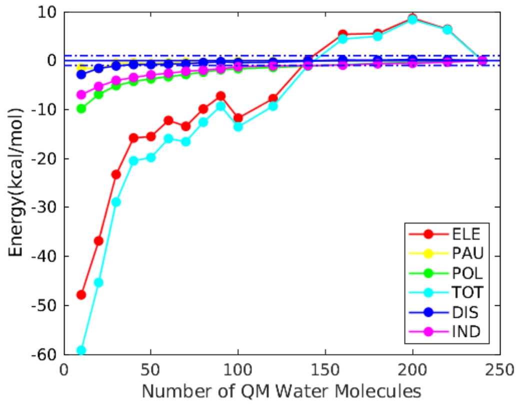

(f)

Figure S1: Interaction energy components in kcal/mol (M062X/cc-pVDZ) relative to the limit value for a QM region with 240 water molecules ($E^{240}_{i,QM/MM}$ and $E^{240}_{i,QM}$) for different sizes of the QM region within an MM (TIP3P) electrostatic embedding (left) and without it (right). Ammonium (top), glycine (middle) and formate (bottom). Horizontal dashed lines: deviation of ±1kcal/mol from E^{240}_{i}, where i represents each component of the interaction energy. Vertical lines: point of convergence of all the energy components within the ±1kcal/mol range.

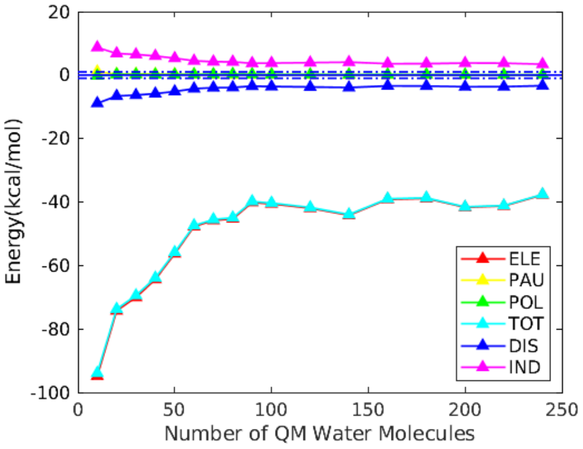

(a)

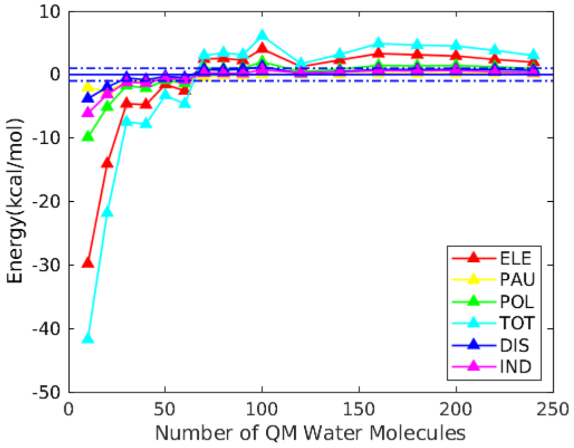

(b)

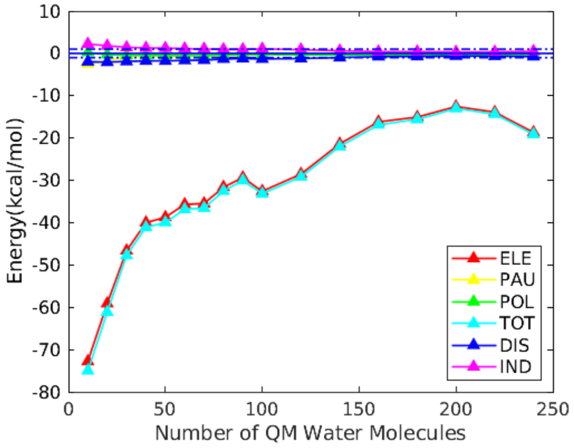

(c)

Figure S2: Deviation for each QM/MM interaction energy component in kcal/mol with respect to the QM energy for QM regions of equal size ($E_i^{j,QM/MM} - E_i^{j,QM}$) for the cationic (a), zwitterionic (b) and anionic (c) solutes. Where i represents each component of the interaction energy and $j = \{10, 20, ..., 240\}$.Total and electrostatic components are not represented for both ions for scale reasons. Horizontal dashed lines: deviation of ±1kcal/mol.
